# Computational approach to evaluate scRNA-seq data quality and gene body coverage with SkewC

**DOI:** 10.1016/j.xpro.2022.102038

**Published:** 2023-01-18

**Authors:** Imad Abugessaisa, Akira Hasegawa, Shintaro Katayama, Juha Kere, Takeya Kasukawa

**Affiliations:** 1 Laboratory for Large-Scale Biomedical Data Technology, RIKEN Center for Integrative Medical Sciences, 1-7-22 Suehiro-cho, Tsurumi-ku, Yokohama City, Kanagawa 230-0045, Japan; 2 Folkhälsan Research Center, Topeliuksenkatu 20, 00250 Helsinki, Finland; 3 Department of Biosciences and Nutrition, Karolinska Institutet, 141 83 Huddinge, Sweden; 4 Stem Cells and Metabolism Research Program, University of Helsinki, P.O. Box 4 (Yliopistonkatu 3), Helsinki, Finland; 5 Institute for Protein Research, Osaka University, Suita, Osaka 565-0871, Japan

**Keywords:** Bioinformatics, Single Cell, RNAseq

## Abstract

SkewC is a single-cell RNA sequencing (scRNA-seq) data quality evaluation tool. The approach is based on determining gene body coverage, and its skewness, as a quality metric for each individual cell. SkewC distinguishes between two types of single cells: typical cells with prototypical gene body coverage profiles and skewed cells with skewed gene body coverage profiles. SkewC can be used on any scRNA-seq data as it is independent from the underlying technology used to generate the data.

For complete details on the use and execution of this protocol, please refer to Abugessaisa et al. (2022).^1^

## Highlights

• SkewC is a data quality tool that distinguishes typical from skewed cells
• SkewC can assess the skewness of scRNA-seq gene body coverage
• Skewed cells are of poor quality and negatively impact downstream analysis
• SkewC can be used on any scRNA-seq data set

## Abstract (editor highlights)

Publisher’s note: Undertaking any experimental protocol requires adherence to local institutional guidelines for laboratory safety and ethics.

## Graphical abstract

**Figure:**
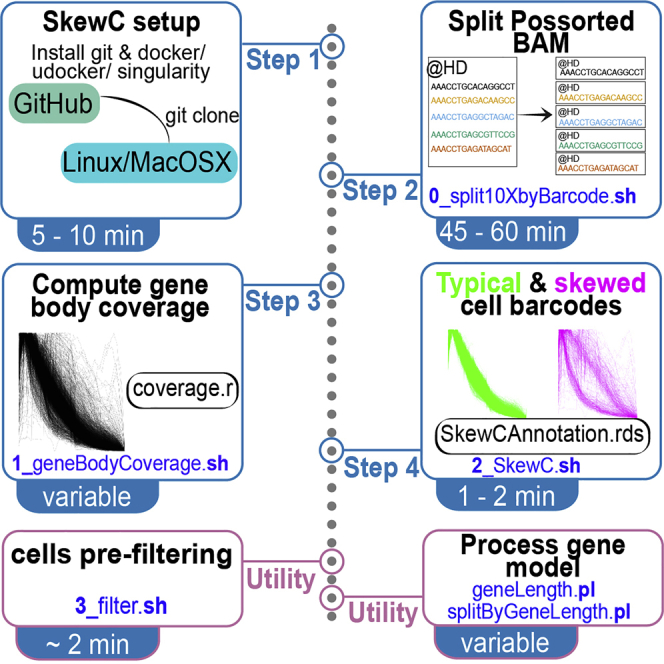

## Before you begin

We present a computational protocol that describes the technical details for the execution of SkewC, a quality assessment tool for scRNA-seq datasets. SkewC measures the quality of each single cell using gene body coverage and its skewness as quality metrics. The distribution of matched sequences throughout the whole gene (5′ to 3′ end) is used to determine the relative gene body coverage, a critical metric to assess the quality of scRNA-seq datasets.^1^ Using skewness of gene body coverage, SkewC defines two types of cells: typical cells (with prototypical gene body coverage) and skewed cells (with skewed gene body coverage). Because of the molecular characteristics of skewed cells and their impact on downstream analysis, SkewC recommends excluding cells with skewed gene body coverage from downstream analysis of scRNA-seq datasets.^1^ SkewC is implemented in the R and Perl computational languages. To enable operability, portability, and ease-of-use, the workflow is provided as a docker (and udocker) container and as a Singularity Image File (SIF) downloadable from SkewC GitHub repository here. SkewC can process any type of scRNA-seq datasets, (full length sequence, 5` -end and 3` -end target protocols).

In our previous publications^3^^,^^4^ we demonstrated the implementation, integration, and utilization of SkewC in scRNA-seq data analysis.

### Input to SkewC

**Figure 1:**
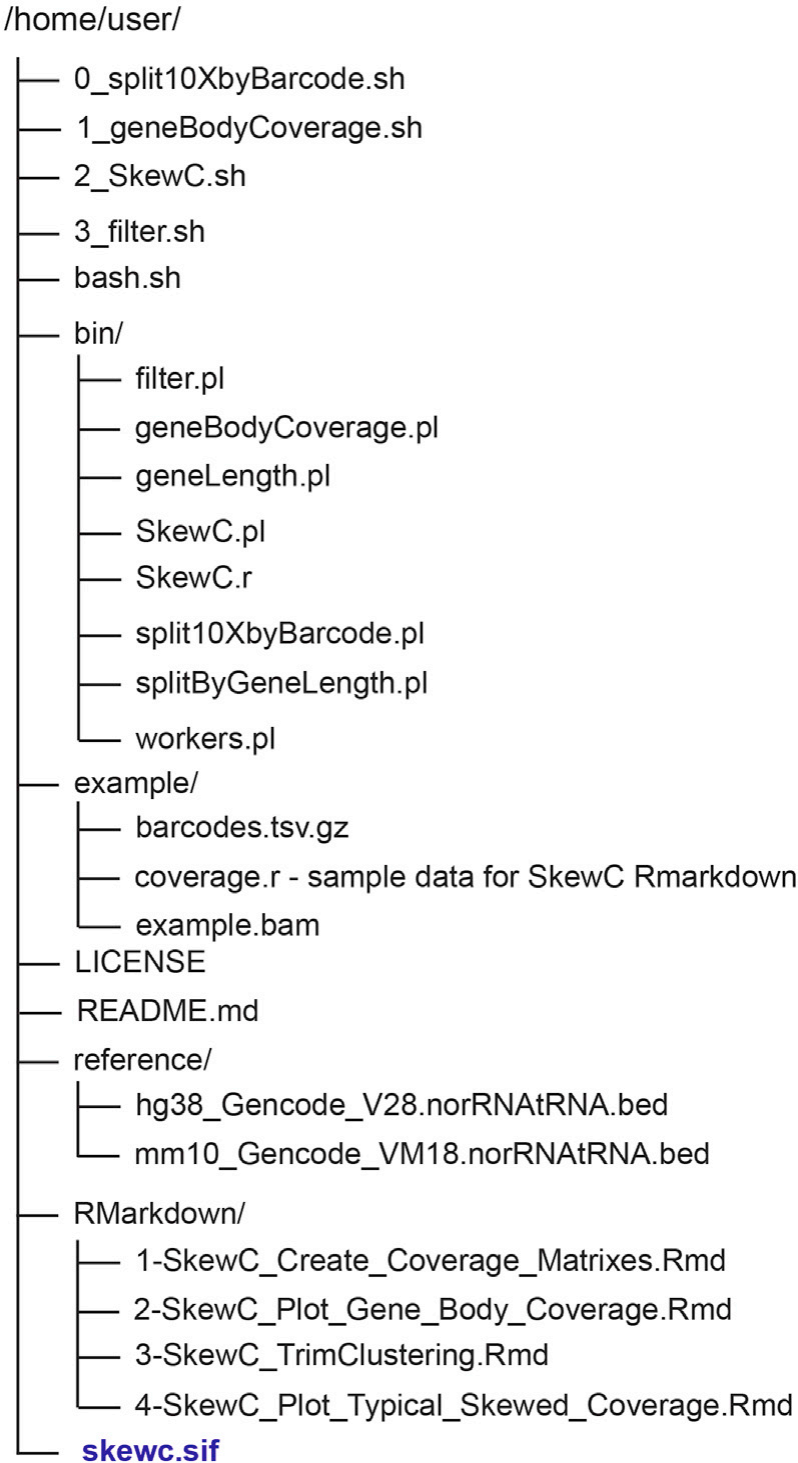
SkewC folder structure under user home directory This structure is created when SkewC is successfully cloned from GitHub.

Based on the source of the scRNA-seq dataset, SkewC accepts different types of input files. Here we describe the accepted inputs to SkewC and their formats and specifications:

1. Biological sequences aligned to a reference genome in BAM format.
  In next generation sequencing, raw sequence reads generated by high throughput sequencers are mapped to the target reference genome assembly using any of the available RNA-seq aligners (e.g., STAR Aligner,^5^ Burrows-Wheeler Aligner,^6^ Salmon^7^ etc.). All aligners generate aligned reads files in Sequence Alignment/Map Format SAM / BAM format.^8^
  a. 10x Genomics provides Cell Ranger, a fully integrated pipeline for alignment of the raw sequence reads to the reference genome and automated analysis of the datasets generated with the 10x Genomics chromium instrument. SkewC accepts the barcoded BAM file (possorted_genome_bam.bam) generated by the Cell Ranger *count* command and usually saved under the *outs* folder. The barcoded BAM file consists of the aligned reads for all the individual cells (barcodes). SkewC will split the barcoded BAM into multiple BAM files (one BAM file per cell barcode). To read more about the content and specifications of the barcoded BAM files refer to 10x Genomics support portal.
  b. For scRNA-seq datasets generated by protocols other than 10x Genomics (e.g., Smart-seq, STRT, etc.), the set of BAM files produced by the read alignment tools should be stored in one folder and provided as an input to SkewC (one BAM file per cell). SkewC accepts both sorted and unsorted BAM file. SkewC will use the BAM file name as cell ID for analysis and annotation of individual cells.

2. Gene Model in BED format.
  SkewC provides custom gene model files in BED (Browser Extensible Data) format^9^ for both human and mouse genomes, but the user it is also able to supply their own gene model files in BED format. Currently SkewC provides two BED files originally downloaded from GENCODE^10^:
  a. hg38_Gencode_V28.norRNAtRNA.bed for human genome.
  b. mm10_Gencode_VM18.norRNAtRNA.bed for mouse genome.
    The BED files are stored in the reference folder contained in the SkewC directory structure (Figure 1).
  c. Fetching latest version of gene model.
    The latest gene model BED files for human can also be fetched from the table browser of the UCSC genome browser^11^ using the following parameters: [clade]: Mammal; [genome]: Human; assembly: Dec. 2013 (GRCh38/hg38); [group]: Genes and Gene Predictions; [track]: ALL GENCODE V41; [table]: Basic (wgEncodeGencodeBasicV34); [output format]: BED - browser extensible data].
  d. To fetch the tRNA and rRNA BED files from UCSC table browser, follow these instructions:
    i. Select the appropriate genome and assembly.
    ii. Select "Variation and Repeats" for group.
    iii. For rRNA: Click the filter button and type "rRNA" for repClass and click "submit".
    iv. For tRNA: Click the filter button and type "tRNA" for repClass and click "submit".
    v. For rRNA and tRNA combined: Click the filter button and type "rRNA OR tRNA" for repClass and click "submit".
    vi. Click “get output”.
    vii. Click “get BED”.
    viii. To remove tRNA and rRNA sequences from the reference, use the intersectBed tool^12^ command:
      intersectBed -split -v -s -wa -a hg38_Gencode_V34.bed -b hg38_rRNA_tRNA.bed > hg38_Gencode_V34.norRNAtRNA.bed

3. Text file with cell IDs / cell barcodes.
  a. To process 10x Genomics datasets, SkewC requires the barcoded BAM file as described above and a text file with cell barcodes (barcodes.tsv) which is usually found under the *filtered_feature_bc_matrix* folder, in the output of the Cell Ranger count pipeline. The number of barcodes in barcodes.tsv should be equal to the number of barcodes in the barcoded BAM file. To read about the barcode text file specifications refer to 10x Genomics support portal
  b. SkewC batch command (3_filter.sh) enables the user to filter cells prior to running SkewC. The 3_filter.sh command is used when the user utilizes another QC method prior to running SkewC (e.g., Seurat QC metrics, see the workflow in^3^). A text file (.txt) with unwanted cell IDs / barcodes is required to run (3_filter.sh). Each matching cell ID / barcode must be in a single line.

### SkewC output

4. SkewC provides several output files:
  a. Plots of the gene body coverages.
    i. Full gene body coverage plot and the mean coverage plot for all cells/barcodes.
    ii. Full gene body coverage plot for typical cells/barcodes.
    iii. Full gene body coverage plot for skewed cells/barcodes.
  b. Two text files with a list of cell ID/cell barcodes and their annotation (typical /skewed) by SkewC.
  c. SkewC output formats.

The output files from running SkewC are also provided in a convenient html file. The html page displays the plots in PDF format and enables download of the resulting SkewC annotation files as both text and R data object (.RDS). Examples of the sample outputs provided by the SkewC pipeline can be found here.

## Key resources table

**Table:** 

REAGENT or RESOURCE	SOURCE	IDENTIFIER
**Software and algorithms**		

Git	Free and open source by Git community	https://git-scm.com/
Docker	Docker Inc.	https://www.docker.com/
Udocker	Gomes et al.^13^	https://github.com/indigo-dc/udocker
Singularity	Contributors to the Apptainer project	https://apptainer.org/
R language	Dessau et al.^14^	https://cran.r-project.org/bin/macosx/ (macOS) https://cran.r-project.org/bin/windows/base/(Windows) https://cran.r-project.org/bin/linux/ (Linux)
RStudio	RStudio, PBC	https://www.rstudio.com/

**Other**		

Operating system	Linux	https://www.linux.org/
Operating system	MacOSX	https://www.apple.com
SkewC runs on desktop environments with high RAM> 32 GB. But it’s recommended to run SkewC in a cluster environment	Abugessaisa et al.^1^	Zenodo https://doi.org/10.5281/zenodo.7475753
Gene annotation for human	Frankish A, et al. GENCODE 2021. Nucleic Acids Res. 2021 Jan 8;49(D1):D916-D923. https://doi.org/10.1093/nar/gkaa1087. PMID: 33270111; PMCID: PMC7778937.	https://www.gencodegenes.org/human/
Gene annotation for mouse	Frankish A, et al. GENCODE 2021. Nucleic Acids Res. 2021 Jan 8;49(D1):D916-D923. https://doi.org/10.1093/nar/gkaa1087. PMID: 33270111; PMCID: PMC7778937.	https://www.gencodegenes.org/mouse/
Dataset used for SkewC development and testing	Abugessaisa et al.^2^	https://single-cell.riken.jp/

## Step-by-step method details

### SkewC setup and testing

**Timing: 10–20 min**

To setup SkewC, the user needs to carefully follow the instructions and implement the following steps:

1. Get the Git (version control system) source and version compatible with your operating system.
  a. Test the installation of Git by using the git command.
    $ git --version
  b. In case Git is not installed, follow the instructions from here to install Git.

2. Install docker/udocker/singularity (key resources table). We give the user three options, but the user’s choice is dependent on the computing environment and user permissions on the machine (admin / ordinary user).
  a. Install docker: If you are installing SkewC to your personal computer and have admin authority, we recommend installing docker.
  b. Install udocker: if you want to run the pipeline in a Linux environment where you don’t have any admin authority (and can’t run docker).
  c. Install singularity: currently singularity more recommended than udocker.
3. Install SkewC: after completing the installation of git and any of docker/udocker/singularity, the user will be able to install SkewC using a single command in the terminal:

$ git clone https://github.com/LSBDT/SkewC.git

***Note:*** After cloning SkewC, a new folder (SkewC) will be created under the user’s home directory with the structure seen in (Figure 1).

**CRITICAL:** If singularity installed as container in step 2, the next step after completing the installation of singularity is to build the Singularity Image File (SIF).

4. To build SIF from a docker image stored in docker hub, please use this command:

**Figure 2:**
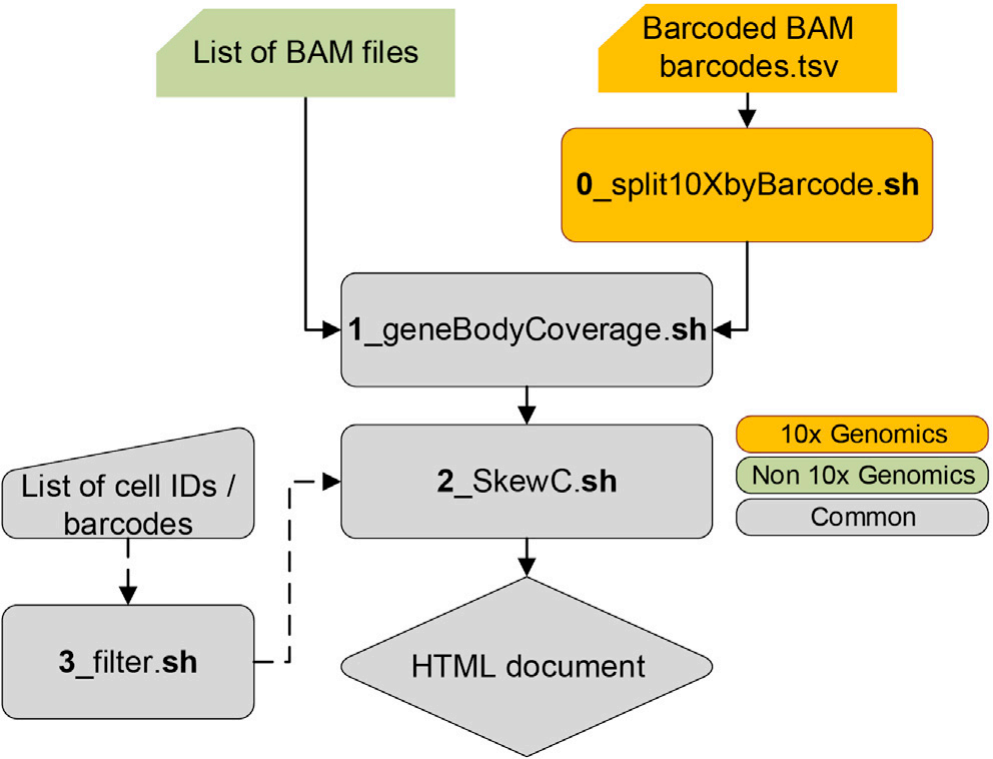
Typical workflow for running SkewC on scRNA-seq data Two types of input are accepted by SkewC. 1) Barcoded BAM files together with the barcodes.tsv file generated by the 10x Genomics protocol. 2) List of BAM files generated by non 10x Genomics scRNA-seq protocols. The output of SkewC is provided in html format with multiple plots and text files. The yellow-colored boxes are for 10x Genomics dataset, green-colored box for non 10x Genomics, gray-colored box for all datasets.

$ cd SkewC.

∼/SkewC$ singularity build skewc.sif docker://moirai2/skewc:latest.

***Note:*** The skewc.sif file will be added to the SkewC work directory (Figure 1). Check the existence of the SIF file built under the work directory of SkewC before continuing with the protocol.

***Note:*** All the subsequent batch commands are executed from the SkewC root directory as outlined in (Figure 2).

5. Testing SkewC; To demonstrate the implementation of SkewC, we provide users with three types of test datasets under the directory ∼/SkewC/TestData. Under the TestData folder there are three subfolders:
  a. To test SkewC with the 10xGenomics (Neurons_900) dataset, use the following commands:
    $ cd SkewC/
    ∼/SkewC$ bash 0_split10XbyBarcode.sh TestData/10xGenomics/barcoded.bam TestData/10xGenomics/barcodes.tsv.gz
    ∼/SkewC$ bash 1_geneBodyCoverage.sh mm10
    ∼/SkewC$ bash 2_SkewC.sh
  b. To test SkewC with the non10x (E-MTAB-2600) dataset, use the following commands:
    $ cd SkewC/
    ∼/SkewC$ bash 1_geneBodyCoverage.sh mm10
    ∼/SkewC$ bash 2_SkewC.sh
  c. To test SkewC with a pre-computed gene body coverage file, use the following commands:
    $ cd SkewC/
    ∼/SkewC$ bash 2_SkewC.sh TestData/coverage.r

### Split barcoded BAM file

**Timing: 45–60 min**

6. Split the barcoded BAM file into a set of BAM files based on the list of barcodes provided in the (barcode.tsv) file. This is achieved using the following command:

∼/SkewC$ bash 0_split10XbyBarcode.sh $bam $barcode $outdir

Arguments:

$bam – BAM file from 10Xgenomics analysis.

$barcode – barcodes.tsv.gz under 10Xgenomics outs/filtered_feature_bc_matrix/.

$outdir – directory to store split BAM files (default = ’input’).

**CRITICAL:** This step is required for scRNA-seq datasets generated by 10x Genomics.

***Note:*** If you don’t wish to designate a specific output directory, you can omit the $outdir argument.

The bash script 0_split10XbyBarcode.sh will create multiple BAM files (one BAM file per cell barcode) under the specified output directory (default = 'input').

### Compute gene body coverage

Time (The time depends on the number and size of the BAM files).

A critical step in SkewC is the gene body coverage computation. This step will enable computation of the gene body coverage for each BAM file (cell). The bash script 1_geneBodyCoverage.sh is used for the gene body coverage computation. Another alternative to use Perl command.

7. Run the gene body coverage bash script:

∼/SkewC$ bash 1_geneBodyCoverage.sh $species $indir $outdir

Arguments:

$species - human 'hg38' or mouse 'mm10' (default = 'hg38′).

$indir - directory where split BAM and index files are stored (default = 'input').

$outdir - directory to store geneBodyCoverage.pl output files (default = 'coverage').

8. Compute gene body coverage through the command line using Perl:

Perl bin/geneBodyCoverage.pl -o coverage reference/hg38_Gencode_V28.norRNAtRNA.bed input/example.TTTGTCATCTAACGGT-1.bam > coverage/example.TTTGTCATCTAACGGT-1.log

***Note:*** geneBodyCoverage.pl will create an index file under a reference directory (default = 'reference') at the beginning of the first iteration. From the second iteration onwards, indexed reference files will be used to speed up calculation.

***Note:*** It’s not recommended to run geneBodyCoverage.pl in parallel when it's creating an index file.

***Optional:*** Parallelization is available on SkewC by running multiple “1_geneBodyCoverage.sh” scripts in parallel. By calling five shell scripts, each script calculates gene body coverage of separated BAM files.

./1_geneBodyCoverage.sh &

./1_geneBodyCoverage.sh &

./1_geneBodyCoverage.sh &

./1_geneBodyCoverage.sh &

./1_geneBodyCoverage.sh &

### Analysis of the gene body coverage and output preparation

**Timing: 1–2 min (for steps 9 and 10)**

The final step in a typical SkewC workflow is the analysis of the gene body coverage and the preparation of the output folder.

9. Run bash script 2_SkewC.sh to analyze gene body coverage.

∼/SkewC$ bash 2_SkewC.sh $prjname $indir $outdir $alpha

Arguments:

$prjname - project name of sample (default = 'COV'). $prjname will be printed on PDF outputs.

$indir - a directory where geneBodyCoverage.pl output files are stored (default = 'coverage').

$outdir - a directory to store skewc analysis files with index HTML (default = 'skewc').

$alpha - alpha for tclust computation with three modes:

(Not defined) - alpha value is decided by highest value from ctlcurves.

1.0–1.0 - tclust will be computed with this user specified value.

10. Use of the alpha value in SkewC, please see the detailed description in the SkewC original publication.^1^ Here are the three possible values for alpha.
  a. bash 2_SkewC.sh test input output - *tclust computation with auto alpha value*.
  b. bash 2_SkewC.sh test input output 0.1 - *tclust computation with alpha = 0.1*.
  c. bash 2_SkewC.sh test input output 0.1 0.2 0.3 0.4 - *tclust computation with alpha = 0.1, 0.2, 0.3, 0.4*.

### Pre-filtering of the cells

**Timing: 2–5 min**

11. Run the bash script 3_filter.sh as follows:

∼/SkewC$ bash 3_filter.sh $filter $indir $matchdir $unmatchdir

Arguments:

$filter - Filter file with list of IDs.

$indir - Input directory (Default = coverage).

$matchdir - match directory with filter list (Default = match).

$unmatchdir - unmatch directory with filter list (Default = unmatch).

***Optional:*** This step is executed only when a user needs to filter out certain cells. The user will need to prepare a text file with a list of cellIDs/barcodes that will be removed from SkewC computation. Example of a list of cell IDs (ERR1211178, ERR1211176, and ERR1211180).

12. After filtering out unwanted cells with '3_filter.sh', run '2_SkewC.sh' again whilst specifying $indir as follows:

∼/SkewC$ bash 2_SkewC.sh $prjname $indir $outdir $alpha

### R Markdown files description

The bash script 2_SkewC.sh utilizes four R Markdown files. These files can either be run within 2_SkewC.sh or in the RStudio environment. Here we are going to describe these R Markdowns in more details. The four R Markdown files are available from SkewC GitHub repository here.

### SkewC_Create_Coverage_Matrix.Rmd

This R Markdown creates the coverage matrix. The input for this file is the vector of normalized values which was created by bash script 1_geneBodyCoverage.sh and stored in the coverage.r. In this file, each single cell has a vector of numerical values (n = 100), and each cell has a cell id / barcode as identifier. The result of running the SkewC_Create_Coverage_Matrix.Rmd is a set of R data frames. After initializing some variables, the script reads the coverage.r file and converts it to the R data frame Coverage_DF. The Coverage_DF data frame consists of 101 columns with each row in the data frame representing the gene body coverage of a single cell. The Coverage_DF is used to compute the mean coverage matrix (Coverage_means_DF). The data frame name Coverage_means_DF consists of 10 columns [pmean10...pmean100] plus the cell ID/ barcode column "Annotation". The Coverage_means_DF data frame is processed to generate the data frame Coverage_means_DF_Clust.

### SkewC_Plot_Gene_Body_Coverage.Rmd

**Figure 3:**
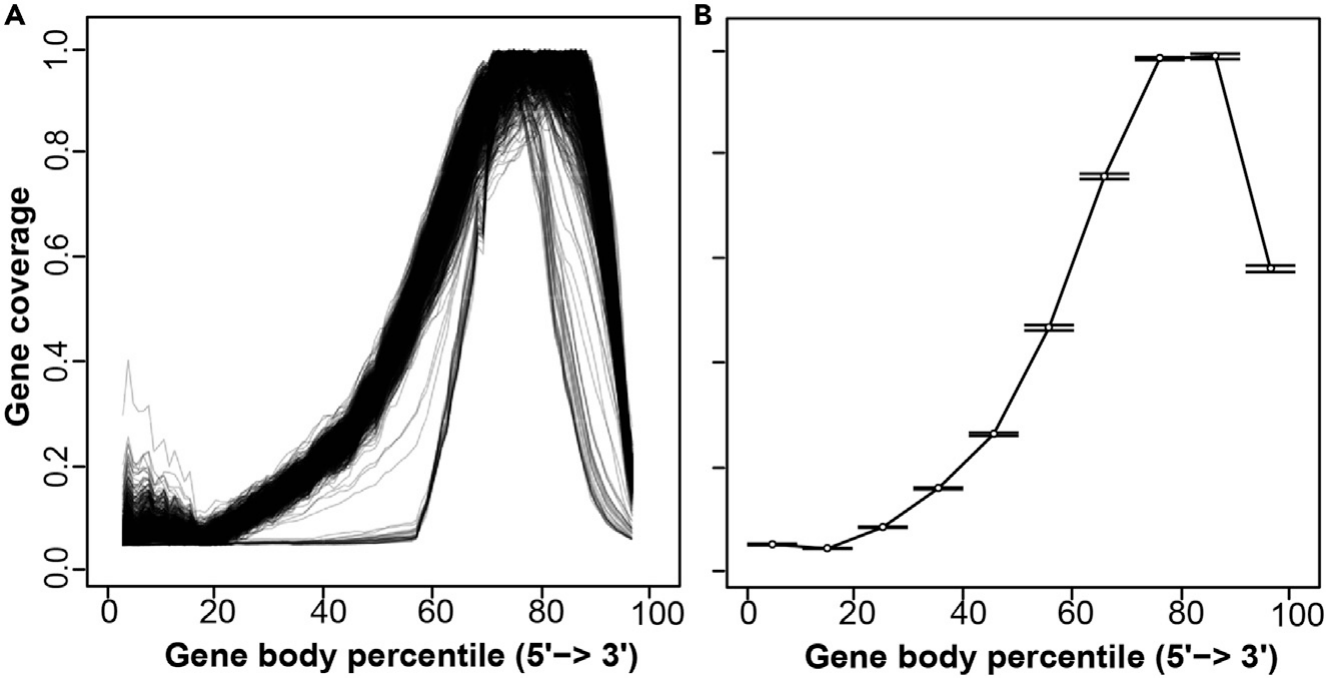
SkewC gene body coverage generated by 2-SkewC_Plot_Gene_Body_Coverage.Rmd (A) The full gene body coverage for the single cell dataset 1k Brain Cells from an E18 Mouse (n = 930). (B) The mean gene body coverage for the single cell dataset 1k Brain Cells from an E18 Mouse (n = 930).

This R Markdown uses the R data frame Coverage_DF (output from SkewC_Create_Coverage_Matrix.Rmd) to generate two types of plots: The Full gene body coverage plot and the mean coverage plot (Figure 3).

### SkewC_TrimClustering.Rmd

**Figure4:**
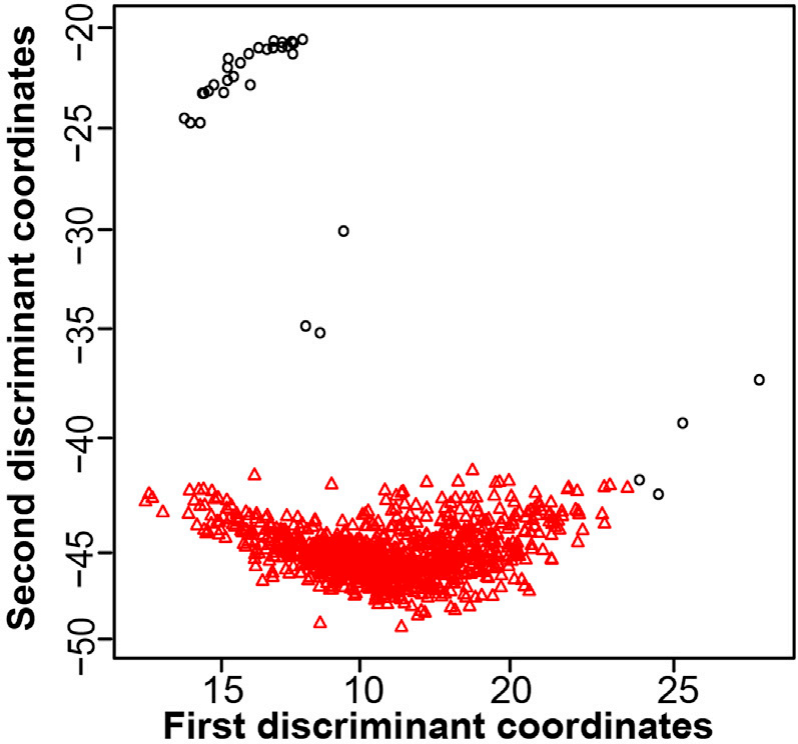
Clustering result for gene body coverage of 1k Brain Cells from an E18 Mouse (n = 930) dataset generated by R tclust function implemented in 3-SkewC_TrimClustering.Rmd R Markdown file Red circles represent typical cells, and the black circles represents skewed cells. Parameters used are k = 1 and α = 0.04.

The R Markdown SkewC_TrimClustering.Rmd performs the trim clustering implemented in R tclust function.^15^ The input to this R Markdown is the R data frame Coverage_means_DF_Clust and alpha value. SkewC enables the user to either select the alpha value or SkewC will auto approximate the optimal trimming level for alpha (please see^1^). The output of this R Markdown is two text files (.tsv), one for the list of typical cells and one with the list of skewed cells. This R Markdown generates a plot CLUSTResult which shows the clustering result of tclust (Figure 4).

### SkewC_Plot_Typical_Skewed_Coverage.Rmd

**Figure 5:**
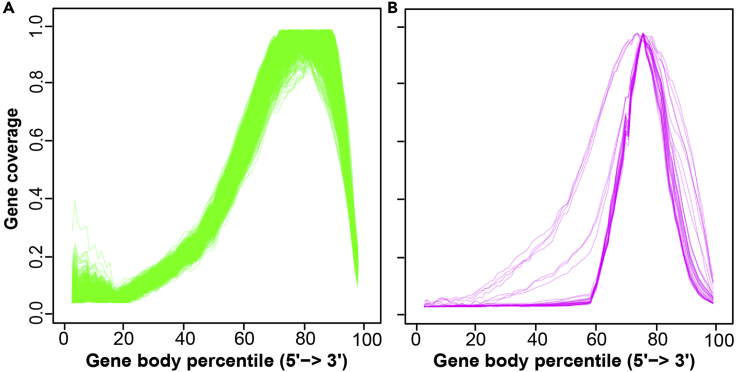
Gene body coverage output of SkewC (typical and skewed cells) SkewC gene body coverage for typical cells (n = 897:930) (A) and skewed cells (n = 33: 930) (B) for the single cell dataset 1k Brain Cells from an E18 Mouse (n = 930). The plots are generated using the R ggplot() function implemented in 4-SkewC_Plot_Typical_Skewed_Coverage.Rmd.

This R Markdown uses the output from SkewC_TrimClustering.Rmd (list of IDs from typical and skewed cells) and the R data frame Coverage_DF to plot two plots: the gene body coverage for the typical cells and the gene body coverage of the skewed cells (Figure 5).

## Expected outcomes

Running SkewC will result html file contains all outputs. An example of SkewC html output is here and here.

As mentioned, SkewC provides visualizations of the gene body coverage for all cells (Figure 3), clustering of the cells based on the gene body coverage (Figure 4) and gene body coverage plots for typical and skewed cells (Figure 5). In addition to the plots, SkewC provides two text files (.tsv) with the list of cell ID / cell barcodes and the SkewC annotation (typical / skewed). The content of the text files is also provided as R data objects (.rds).

***Note:*** The SkewC annotation can be added as a metadata column to R SingleCellExperiment class / R Seurat object / python anndata for further analysis or filtering of cells during an scRNA-seq data analysis workflow.

## Limitations

Gene body coverage is a computationally demanding task and users of SkewC may have concerns about the computation time that is required for scRNA-seq datasets containing thousands of cells.

• We conducted performance evaluation of SkewC in.^1^ We tested SkewC using the public dataset published by 10x Genomics “pbmc_8k” which contains a total of 8,000 cells. We split the post-processed BAM file into 6 files, each one containing different amounts of cells as shown in the x-axis of Figure 7 in.^1^ We ran SkewC four times, for each of the BAM files containing different numbers of cells (using 10 cores). As an example, running SkewC on a BAM file containing 500 cells took on average 3 h to compute the gene body coverage.
• It’s important to mention that the performance depends both on the BAM file and reference genome size in GB.
• Performance evaluation analysis of SkewC shows a linear association between the number of cells and SkewC runtime.
• Users of SkewC will be able to increase the number of cores to reduce the computation time, depending on the availability of resources.

## Troubleshooting

### Problem 1

Failed to run git command.

Failed to run ∼/git clone https://github.com/LSBDT/SkewC.git.

The user failed to run the git command to clone SkewC (related to SkewC setup). This problem occurs when git was not installed in the computing environment. Other problems related to the installation and cloning of SkewC is that either docker, udocker and singularity is not installed on the user’s system.

### Potential solution

To overcome this problem, the user needs to follow the instructions Git support to install the latest version of Git. Then follow all steps in SkewC setup.

### Problem 2

Failed to open SkewC singularity image file (sif):

The error message “*Could not open image ∼/SkewC/skewc.sif: failed to retrieve path for ∼/SkewC/skewc.sif: lstat ∼ /SkewC/skewc.sif: no such file or directory*”.

This problem related to step 4 in SkewC setup ; the above error will appear when a user tries to run the first batch command 0_split10XbyBarcode.sh after cloning SkewC.

### Potential solution

• The user needs to check that the skewc.sif file was built and is located under the work directory of SkewC (step 4 in SkewC setup).
• If skewc.sif is missing, the user needs to build the SIF file. To build SIF from the docker image stored in the docker hub, please refer to SkewC setup.

### Problem 3

Perl: warning: Setting locale failed.

The following warning messages will not stop the execution of SkewC, and the script will run as intended (related to split barcoded BAM files and compute gene body coverage).

“*perl: warning: Setting locale failed.*

Perl: warning: Please check that your locale settings:

LANGUAGE = (unset),

LC_ALL = (unset),

LANG = “en_US.utf-8”

are supported and installed on your system.

*Perl: warning: Falling back to the standard locale (“C”)*”.

The above Perl warnings appear when running the batch command bash 0_split10XbyBarcode.sh $bam $barcode $outdir. The warning is related to the local environment settings. The warning will not impact the finalresults of the batch command.

### Potential solution

Users of SkewC need to confirm the local settings (language installation) as recommended in the warning message.

### Problem 4

Perl: Error: package or namespace load failed for 'reshape2' in dyn.load(file, DLLpath = DLLpath, ...).

The following error message may appear “*package or namespace load failed for ‘reshape2’ in dyn.load(file, DLLpath = DLLpath, …): unable to load shared obje’t '/home/imad-a/R/x86_64-pc-linux-gnu-ibrary/3.4/stringi/libs/stringi’so': libicui18n.so.57: cannot open shared object file: No such file or directory*.

*Execution halted*.

*No alpha value computation found*”.

The above error message (related to analysis of the gene body coverage and output preparation).

This error will not allow completion of SkewC execution and will terminate the process. This error is related to the R statistical packages used in the R markdown files described above; the error is not fixable from the user’s side.

### Potential solution

Users should run git clone step 3 in skewc setup and testing again to ensure that the latest version of SkewC is installed. If the problem persists, the user should contact the SkewC developers to re-build the SkewC container and release a new version of SkewC.

### Problem 5

Empty gene body coverage plot.

This problem relates to (step 1 in split barcoded BAM file). It occurs when there is a mis-match between the barcode names in the barcode.tsv file and the barcodes in the barcoded BAM file. As a result, user will find an empty PDF file of the gene body coverage plot in the $outdir. This output-error occurs after running the bash command described in analysis of the gene body coverage and output preparation steps.

### Potential solution

• Confirm the format of the barcodes in barcode.tsv and the barcoded BAM file match.
• In case of a mismatch, reformatting will be necessary. Use a text editor to change the format in any of the two files. Then re-run (step 1 in Split barcoded BAM file) and the rest of SkewC workflow.

## Resource availability

### Lead contact

Further information and requests for source code and data processing protocols should be directed to, and will be fulfilled by, the Lead contact Takeya Kasukawa (takeya.kasukawa@riken.jp).

### Materials availability

This study did not generate new unique reagents.

## Data Availability

• Full and complete source code of SkewC is freely available for download from SkewC GitHub repository here. • Zenodo https://doi.org/10.5281/zenodo.7475753. • Datasets used during the development of SkewC are available from our scRNA-seq database SCPortalen^2^ here.
